# Investigation of Radiation Effects on FD-SOI Hall Sensors by TCAD Simulations

**DOI:** 10.3390/s20143946

**Published:** 2020-07-16

**Authors:** Linjie Fan, Jinshun Bi, Kai Xi, Gangping Yan

**Affiliations:** 1 Institute of Microelectronics, Chinese Academy of Sciences, Beijing 100029, China; fanlinjie19@mails.ucas.ac.cn (L.F.); xikai@ime.ac.cn (K.X.); yangangping@ime.ac.cn (G.Y.); 2 School of Microelectronics, University of Chinese Academy of Sciences, Beijing 100049, China

**Keywords:** Hall sensors, FD-SOI CMOS, radiation effects, total ionizing dose, transient dose rate, single event transient, 3D TCAD simulations

## Abstract

This work investigates the responses of the fully-depleted silicon-on-insulator (FD-SOI) Hall sensors to the three main types of irradiation ionization effects, including the total ionizing dose (TID), transient dose rate (TDR), and single event transient (SET) effects. Via 3D technology computer aided design (TCAD) simulations with insulator fixed charge, radiation, heavy ion, and galvanomagnetic transport models, the performances of the transient current, Hall voltage, sensitivity, efficiency, and offset voltage have been evaluated. For the TID effect, the Hall voltage and sensitivity of the sensor increase after irradiation, while the efficiency and offset voltage decrease. As for TDR and SET effects, when the energy deposited on the sensor during a nuclear explosion or heavy ion injection is small, the transient Hall voltage of the off-state sensor first decreases and then returns to the initial value. However, if the energy deposition is large, the transient Hall voltage first decreases, then increases to a peak value and decreases to a fixed value. The physical mechanisms that produce different trends in the transient Hall voltage have been analyzed in detail.

## 1. Introduction

Hall sensors are the most common converters used to turn a magnetic field into an electric signal. Owing to their advantages of non-contact, strong anti-interference, high linearity, robustness, and versatility [1], Hall sensors are used in scientific detecting, brushless DC motors, contactless measurements and so on [2,3,4]. Compared to the conventional bulk Si CMOS (complementary metal-oxide-semiconductor) for Hall sensor manufacturing, the choice of fully-depleted silicon-on-insulator (FD-SOI) technology brings several important advantages. The FD-SOI structure not only has the advantages of less noise generation, lower biasing voltage, and higher integration density [5,6,7], but it has also been confirmed that—compared with the bulk structure—the characteristics of the sensors (such as sensitivity and efficiency) are improved because of its thin thickness and low doping concentration [8].

When used in aerospace systems or nuclear weapon control systems, sensors must function normally while being strongly irradiated. In general, the radiation environment that the sensors are exposed to mainly space radiation and man-made nuclear radiation [9]. For instance, there are cosmic rays, Van Allen Belt, and solar flares in the space environment, which may result in total ionizing dose (TID) effects and single event transient (SET) effects on the sensors [10,11]. Moreover, transient and high-energy X-ray and gamma-ray produced by nuclear explosions and dayglow could lead to a transient dose rate (TDR) effect [12]. Therefore, it is necessary to study the radiation effects on the performance of sensors used in harsh radiation conditions.

Previously published literature mainly focused on a single type of radiation effect on the Hall sensor under a certain radiation source, such as gamma [13,14,15], proton [16,17], and neutron radiation [18,19]. Karlova et al. [14] studied the influence of gamma radiation on sensors based on GaAs. However, this research was limited to the analysis of the volt-ampere (IV) characteristics and noise spectral density before and after gamma irradiation. Therefore, the analysis of the effects of irradiation on magnetoelectric properties such as the Hall voltage and sensitivity has not been investigated. Abderrahmane et al. [16] have irradiated GaN-based Hall sensors by protons with the energy of 380 keV and fluence from 10^14^ p/cm^2^ to 10^16^ p/cm^2^. The changes in the electron mobility, sheet resistance, and sensitivity of the sensors before and after irradiation have been observed. Moreover, most published studies focus on III-V semiconductor-based Hall sensors. There are few reports on the radiation effects of silicon-based—especially FD-SOI—Hall sensors aimed at sensor and peripheral circuit monolithic integration [20].

This work investigates the radiation effects on the performance of the FD-SOI Hall sensors with the use of 3D technology computer aided design (TCAD) tools. It focuses on the impacts of radiation effects (such as the TID, TDR, and SET effects) on the performance of the FD-SOI Hall sensors. The figure-of-merits (FOMs) includes the Hall voltage, sensitivity, efficiency, and offset voltage. The remainder of this paper is organized as follows. Section 2 introduces Hall sensor theory, the FD-SOI Hall sensor structure, and the TCAD simulation methodology. Section 3 explores the performance of the FD-SOI Hall sensor under different irradiation effects via physical simulations and discusses the physical mechanism. Finally, Section 4 summarizes the radiation effects of FD-SOI Hall sensors.

## 2. Methodology and Physical Models

### 2.1. Hall Sensor Theory

This section briefly introduces the figure-of-merits (FOMs) definitions for Hall sensor evaluation. The specific relations of the FOMs are shown in Table 1. Moreover, in the following sections, the changes in these parameters during or after radiation will be evaluated.

When the semiconductor with current flowing through is placed in a magnetic field, the carriers in the semiconductor is biased to one side by Lorentz force, and then Hall voltage (*V_H_*) is generated. The sensitivity is one of the most significant FOMs related to a Hall sensor. Generally speaking, the sensitivity is defined as the change in output concerning a given change in input. In addition, a FOM related to the power consumption of the sensor should also be noted. An efficiency factor (*η*), which is also known as power-related sensitivity, indicates how much Hall voltage will be generated by consuming 1 W under the magnetic induction of 1 T. It is well known that even if the sensor is biased at zero magnetic field, there is also a parasitic voltage named the offset voltage (*V_offset_*). The *V_offset_* can be generated by misalignment of contacts, asymmetry of the geometric dimensions and non-uniformity of the active region material [20]. In practical applications, it is obvious that the *V_offset_* should be as small as possible.

### 2.2. Device for Simulation

**Figure 1:**
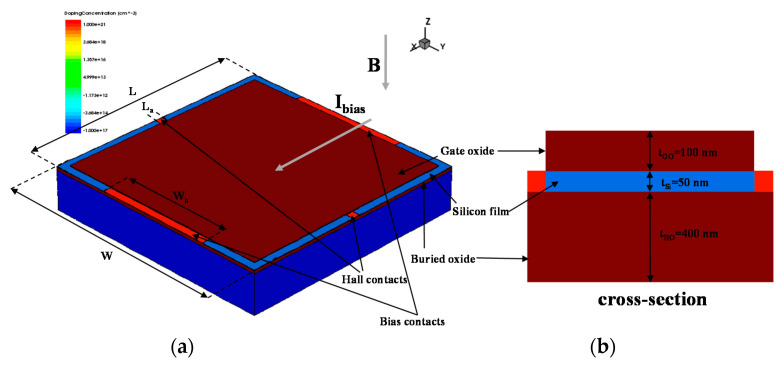
(**a**) 3D geometric model, and (**b**) cross-section of the fully-depleted silicon-on-insulator (FD-SOI) Hall sensor for the technology computer aided design (TCAD) simulation.

The optimal FD-SOI Hall sensor structure has been demonstrated in the authors’ previous work [8]. There is a brief summary in Appendix A. The 3D geometric model and cross-section of the FD-SOI Hall sensor are illustrated in Figure 1. Moreover, the specific geometrical dimensions and doping concentrations of the FD-SOI Hall sensors are included in Table 2. The doping concentration of the 50 nm silicon film above the buried oxide is 1 × 10^16^ cm^−3^, thereby forming the FD state. Four heavily doped contacts are located in the center of the four sides of the silicon film. When a bias current (*I_bias_*) is applied to the bias contacts, the Hall voltage can be detected at the Hall contacts under the magnetic induction intensity (*B*) in the negative direction of the *z*-axis. In particular, by applying a voltage to the gate (*V_g_*) on the gate oxide layer, the sensors can be turned on or off.

### 2.3. Simulation Models

The ionization damages of ionizing radiation in the materials lead to the severe degradation of device performance, triggering three ionizing radiation effects: the TID, TDR, and SET effects.

The TID effect refers to the effect of radiation damage related to time accumulation by X-ray, gamma-ray, and charged particles. Electron-hole (e-h) pairs are generated in the insulator region during the TID radiation. Most electrons are quickly swept out of the oxide under the electric field because of the large mobility of electrons. In this process, some electrons will be recombined with holes. At the same time, holes will also be relatively slowly transported to the Si/SiO_2_ interface under the electric field. Parts of the holes are trapped in the oxide layer, forming a net positive oxide layer trap charge [22]. The final effect is the negative drift of the threshold voltage in n-channel transistor:

(1) $${\Delta V}_{ot}=-\frac{q}{\varepsilon_{ox}}t_{ox}{\;\Delta N}_{ot}$$

where *ε_ox_* is the dielectric constant of the oxide, *t_ox_* is the thickness of the oxide layer, and *ΔN_ot_* is the net positive trap charge density of the oxide layer. In this work, the insulator fixed charge model in Sentaurus TCAD activated in oxide layer is adopted to simulate the above TID effect [23]. 

The TDR effect occurs in the environment of transient large dose radiation, such as with nuclear explosions and solar storms. The TDR radiation deposits a large amount of energy in the semiconductor device, generating a huge number of e-h pairs. Different radiation sources (gamma-ray, X-ray, or electron) lead to different charge generation and recombination rates [24,25,26]. In this work, the main source taken into account for the radiation simulation is gamma-ray, which is also the main method for evaluating the TDR effect of semiconductor devices and integrated circuits. The gamma radiation model in Sentaurus TCAD is used to simulate the TDR effect. The generation of e-h pairs caused by gamma radiation depends on the electric field (*E*), described by:

(2) $$G_{r}{=g}_{0}D\cdot Y(E)$$

where *g*_0_ is the generation rate of e-h pairs, *D* is the dose rate, and *Y*(*E*) is a function related to the electric field. *G_r_* is derived as a linear function of the dose rate.

The SET effect refers to the generation of a large number of e-h pairs along the track of incident high-energy particles hitting a semiconductor device. The electrodes collect the e-h pairs, causing soft errors in the circuits and permanent damage in severe cases [27,28]. The heavy ion model in Sentaurus TCAD has an input parameter linear energy transfer (LET), which describes the capability of energy deposition in case of heavy ions penetrating a semiconductor. The generation rate of the e-h pairs caused by heavy ion incidence is computed by:

(3) $${G(l,w,t)=G}_{LET}\left(l)R(w,l)T(t\right)$$

where *G_LET_*(*l*) is the linear energy transfer generation density, and *R*(*w*,*l*) and *T*(*t*) are the functions describing the spatial and temporal variations of the generation rate, respectively.

The galvanic transport model handles the magnetic field acting on the semiconductor Hall sensor. The galvanic transport model is based on the common drift-diffusion transport model enhanced with magnetic field-dependent terms taking Lorentz force into account. The following equations for holes and electrons govern its behavior:

(4) $$\vec{J_{\alpha }}=\mu_{\alpha }\vec{g_{\alpha }}+\mu_{\alpha }\frac{1}{1+(\mu_{\alpha }^{*}{B)}^{2}}[\mu_{\alpha }^{*}\vec{B}\times \vec{g_{\alpha }}+\mu_{\alpha }^{*}\vec{B}\times (\mu_{\alpha }^{*}\vec{B}\times \vec{g_{\alpha }})]$$

where *α* = *n* or *p*, $\vec{g_{\alpha }}$ is the current vector without mobility, $\mu_{\alpha }^{*}$ is the Hall mobility, $\vec{B}$ is the magnetic field vector, and *B* is the magnitude of the vector $\vec{B}$ [29]. Other than the above-mentioned radiation-related and magnetic-field related models, the TCAD simulation also takes many physical models into account, such as SRH generation/recombination and mobility degradation due to high doping concentration, rough surface scattering, and high field saturation. 

## 3. Experimental Results and Discussion 

In this section, with the support of the TCAD simulation, the effects on the performance of the FD-SOI Hall sensor during and after radiation are evaluated. Based on the simulation results, the physical processes of the radiation effects are analyzed.

### 3.1. TID Effect

**Figure 2:**
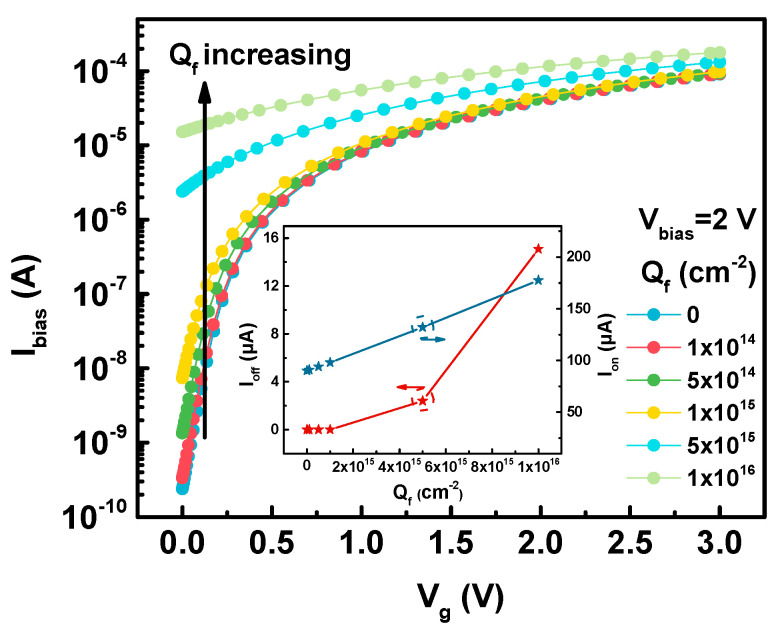
Simulation results of the *I_bias_–V_g_* curve of a FD-SOI Hall sensor with changes in the fixed charge density (*Q_f_*) in the oxide. The inset shows the variation of the leakage current (*I_off_*) and the on current (*I_on_*) with fixed charge density.

As described in Section 2.3, the effect of the TID on the device can be made equivalent by setting the fixed positive charge in the oxide layer. Under low-dose radiation conditions, the trap charge density (i.e., fixed charge density in the simulation) in the oxide induced by radiation is linearly related to the radiation dose, and the trap charge density tends to saturate at medium to high radiation doses [30]. Therefore, different fixed charge densities (*Q_f_*) are equivalent to different radiation doses. The transfer characteristic curve of the FD-SOI Hall sensor with different fixed charge densities is presented in Figure 2. The fixed charge-induced off-state leakage current (*I_off_*, *I_bias_*@*V_g_* = 0 V) and the on-state current (*I_on,_ I_bias_*@*V_g_* = 3 V) are extracted and displayed in the inset.

As shown in Figure 2, an increase in the fixed charge density, which means an increase of the TID radiation, causes an increase in the magnitude of the off-state leakage current. When the charge density reaches 5 × 10^15^ cm^−2^, the off-state leakage current is four orders of magnitude higher than the pre-radiation case, signifying that the sensor loses the capacity of a normal switch. In addition, since the fixed charge affects the threshold voltage, the on-state current also increases as the fixed charge density increases. With the change of *Q_f_* from 0 to 1 × 10^16^ cm^−2^, Ion increases from 90.2 µA to 177.5 µA (an increase of 96.78%).

**Figure 3:**
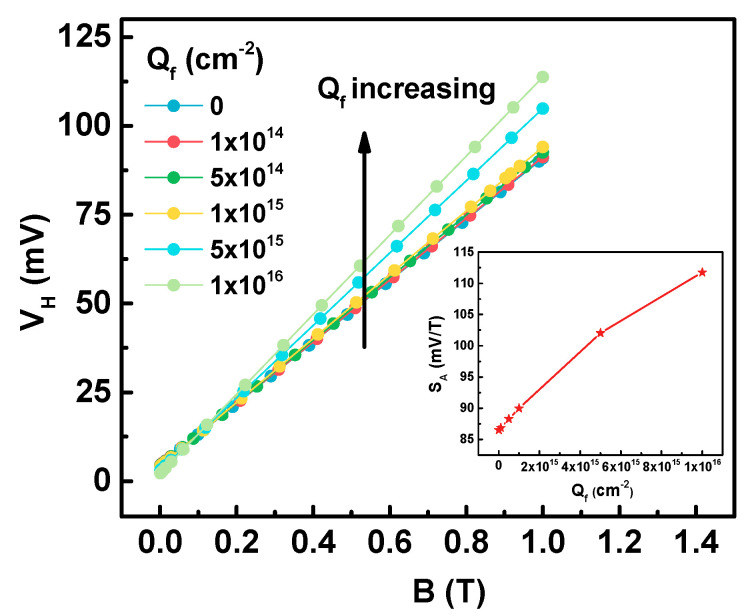
Hall voltage (*V_H_*) versus magnetic induction (*B*) simulated for sensors with different fixed charge densities (*Q_f_*) in the oxide. The inset shows the variation of the absolute sensitivity (*S_A_*) with fixed charge density.

To evaluate the effect of fixed charge on sensor performance, the magnetic induction is swept from 0 T to 1 T with *V_g_* = 3 V and *V_bias_* = 2 V. Figure 3 demonstrates that the Hall voltage increases as the fixed charge density increases. At the same time, by the calculation of equation in Table 1, the absolute sensitivity also increases. The absolute sensitivity increases from 86.49 mV/T to 111.75 mV/T after adding 10^16^ cm^−2^ fixed charge. As expressed in the equation in Table 1, the Hall voltage will increase as the bias current increases. The bias current is increased because of the fixed charge present in the oxide layer, which leads to an increase in absolute sensitivity.

**Figure 4:**
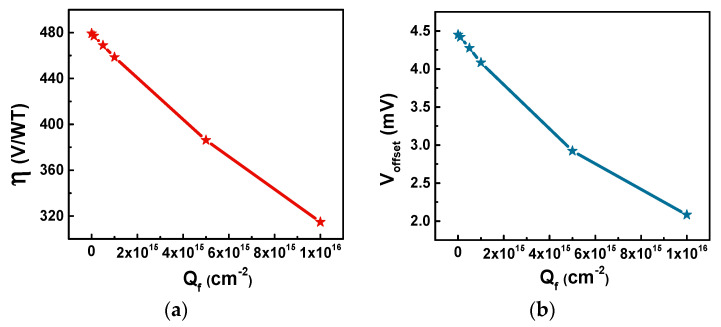
Simulated (**a**) efficiency factor (*η*), and (**b**) offset voltage (*V_offset_*) as a function of fixed charge densities (*Q_f_*).

In addition to absolute sensitivity, efficiency and offset voltage are also extracted, as shown in Figure 4. Both the Hall voltage and the bias current increase because of the existence of a fixed charge, but the bias current growth rate is greater, which therefore leads to a decrease in the efficiency of the sensor. The efficiency of the sensor drops from 479.2 V/WT to 314.7 V/WT after adding 10^16^ cm^−2^ fixed charge, while the offset voltage of the sensor decreases from 4.45 mV to 2.1 mV. The offset voltage is related to the symmetry, uniformity, resistance, and other factors of the sensor [20]. The existence of the fixed charge in the oxide layer causes the changes of the electric field and even the resistivity of the silicon film, so that the offset voltage changes.

### 3.2. TDR Effect

**Figure 5:**
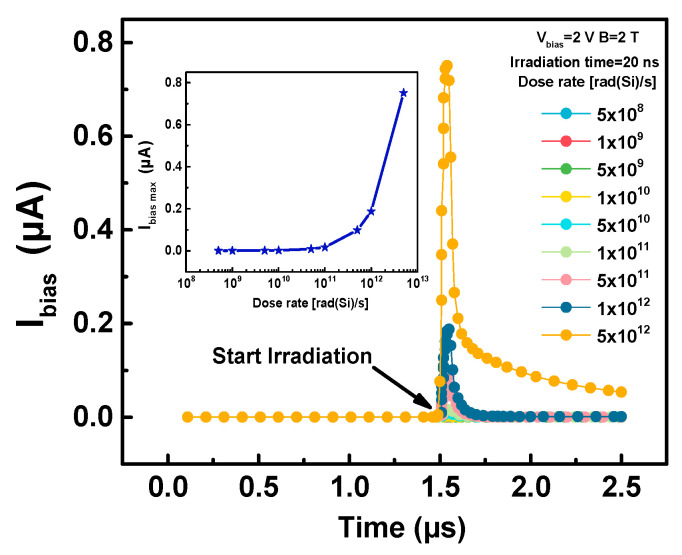
Transient dose rate (TDR)-induced currents in the FD-SOI Hall sensors. The inset shows the change of the detected maximum bias current (*I_bias_*) with dose rate.

The TDR effect investigates the changes in sensor characteristics at the moment of transient radiation like a nuclear explosion. For the TCAD simulation, a high dose rate from 5 × 10^8^ rad(Si)/s to 5 × 10^12^ rad(Si)/s with 20 ns duration is established. Meanwhile, the *B* of 2 T along the negative direction of the *z*-axis is applied. TDR-induced photocurrents under off-state (*V_g_* = 0 V) are detected at *V_bias_* = 2 V, as shown in Figure 5.

As can be observed from Figure 5, at the moment of the start of transient radiation, a current is detected at the bias contact. This is because a large number of e-h pairs are generated at the moment of irradiation, and some e-h pairs that do not have enough time to recombine move under the electric field generated by the bias contacts to form a current. Moreover, as the dose rate increases, the maximum bias current (*I_bias max_*) detected is larger. When the dose rate reached 5 × 10^1^^2^ rad(Si)/s, the detected *I_bias max_* even exceeded 0.5 µA.

**Figure 6:**
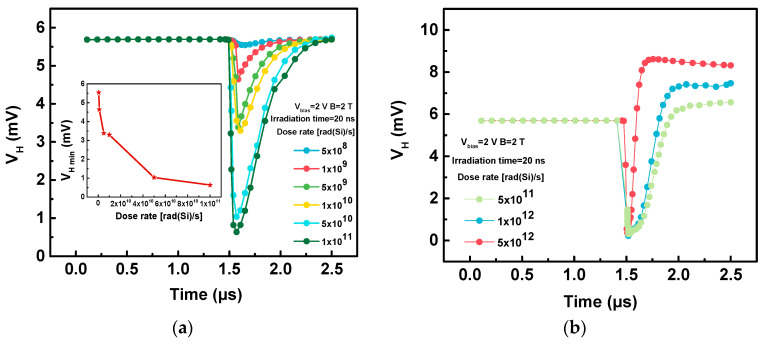
The changes in Hall voltage (*V_H_*) during the irradiation of (**a**) dose rate = 5 × 10^8^~1 × 10^11^ rad(Si)/s and (**b**) dose rate = 5 × 10^11^~5 × 10^12^ rad(Si)/s. The inset in (**a**) is the change in minimum Hall voltage (*V_H min_*) with dose rate.

Figure 6. shows the changes in the Hall voltage during irradiation at different dose rates. Since the sensor is in off-state, the initial Hall voltage value before irradiation is the superposition value of *V_offset_* and Hall voltage formed by *I_off_* under the *B*. On the one hand, when the irradiation dose rate is at a smaller value range (Figure 6a), the Hall voltage will first decrease and then return to the initial value during the irradiation process. At this time, the minimum Hall voltage (*V_H min_*) will decrease as the dose rate increases. On the other hand, when the irradiation dose rate is at a larger value range (Figure 6b), the Hall voltage will rapidly decrease, then increase to reach the maximum value, and finally decrease to a fixed value. At this time, the maximum Hall voltage (*V_H max_*) will increase as the dose rate increases.

**Figure 7:**
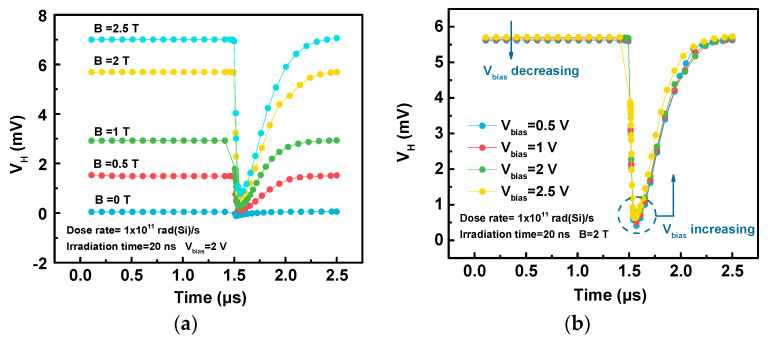
During irradiation with a dose rate of 1 × 10^11^ rad(Si)/s, the variations of Hall voltage (*V_H_*) under different (**a**) magnetic inductions (*B*), and (**b**) bias voltages (*V_bias_*).

From the equation in Table 1, the Hall voltage is related to the bias and magnetic induction. Figure 7 depicts the variations of the Hall voltage during irradiation with a dose rate of 1 × 10^11^ rad(Si)/s under different bias voltages and magnetic inductions. The magnetic induction and bias voltage mainly affect the initial and minimum Hall voltages. The larger the bias voltages and magnetic inductions are, the greater the initial Hall voltage, which will result in a larger built-in electric field. Therefore, the larger built-in electric field creates a greater *V_H min_* and a faster rate at which the Hall voltage returns to its initial value after irradiation.

### 3.3. SET Effect

The SET effect influences the characteristics of a sensor after a single high-energy particle injection. In the TCAD simulation, at the time of 1.52 μs, heavy ions strike in the middle of the sensor along the negative direction of the *z*-axis with the LET ranging from 0 to 100 MeV·cm^2^/mg. The ion trajectories have a Gaussian radial distribution with a characteristic radius of 20 nm. The sensor in the TCAD simulation is biased to off-state (*V_g_* = 0 V) with *V_bias_* = 2 V. Meanwhile, the B of 2 T along the negative direction of *z*-axis is applied.

**Figure 8:**
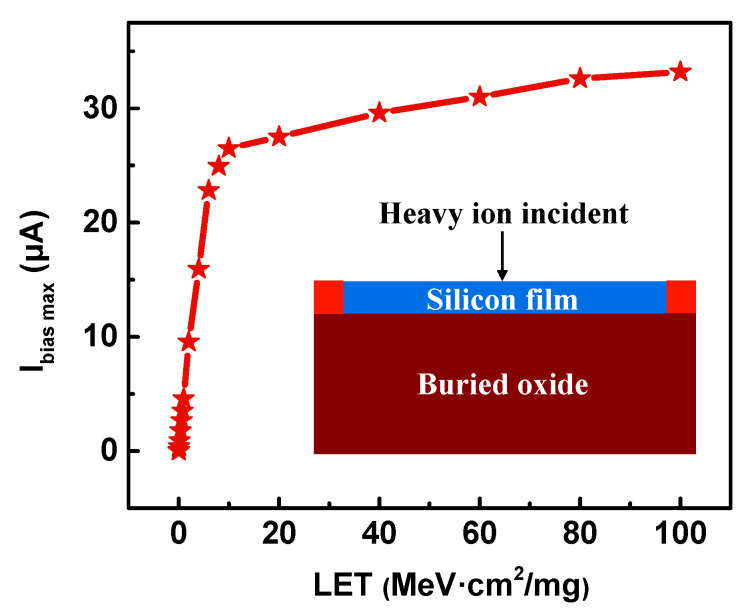
The change of the detected maximum bias current (*I_bias_*) with linear energy transfer (LET). The inset shows a schematic diagram of heavy ion incidence.

After heavy ions enter the sensor, a large number of e-h pairs are generated along the particle track, which are collected by the bias contacts to form the transient current. Moreover, with the increase of the LET, the peak value of *I_bias_* (*I_bias max_*) keeps increasing, as shown in Figure 8. This is because the larger the LET is, the more e-h pairs are generated. So the charge collected by the bias contacts is increased accordingly. Moreover, it can be seen from Figure 8 that when the LET is lower than 10 MeV·cm^2^/mg, the *I_bias max_* increases faster with the increase of LET. Then, after 10 MeV·cm^2^/mg, the increasing trend is gentler. Through fitting, the slopes of the curves before and after 10 MeV·cm^2^/mg are 3.38 μA/(MeV·cm^2^/mg) and 0.077 μA/(MeV·cm^2^/mg), respectively.

**Figure 9:**
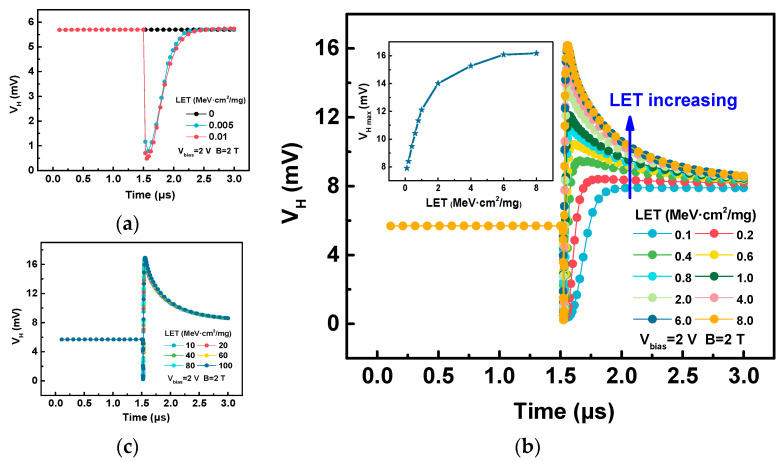
Time variations of the Hall voltages (*V_H_*) with (**a**) low, (**b**) medium, and (**c**) high LETs. The inset in (**b**) is the change in maximum Hall voltage (*V_H max_*) with LETs.

Figure 9 depicts the changes in the Hall voltage with time under different LET ranges. Firstly, when the ions incident with low LETs (Figure 9a), the Hall voltage will first decrease and then return to the initial value. Secondly, when the LETs are at medium values (Figure 9b), the Hall voltage will first decrease, then increase to the maximum value (*V_H_*_*max*_), and finally decrease to a fixed value. It can be observed that the *V_H max_* increases with the increase of the LET, and gradually tends to saturation. Thirdly, when the LETs are in a higher range, the change of the Hall voltage during the heavy ion incidence is basically the same, that is, it decreases rapidly, then increases to the *V_H max_*, and finally decreases to a fixed value.

### 3.4. Summary and Discussion

During or after irradiation, the performance of the FD-SOI Hall sensors, such as the Hall voltage, sensitivity, efficiency, and offset voltage, changes are worthy of investigation. Table 3 summarizes the effects of three types of radiation effects on the FD-SOI sensor performance.

The TID effect mainly explores the changes in the characteristics of the sensor after irradiation. After irradiation, a net positive charge in the oxide layer mainly affects the off-state leakage current and on-state bias current. Since the sensitivity and efficiency of the sensor are both related to the bias current, they will also change after irradiation. At the same time, the holes trapped in the oxide layer affect the resistance of the silicon film of the sensor, leading to the reduction of the sensor offset voltage.

The TDR and SET effects explore the changes of transient current generated and the Hall voltage in the off-state sensor during irradiation. The dose rate in the TDR effect simulation and the LET in the SET effect simulation both determine the irradiation energy deposited on the sensor during the irradiation process. The physical mechanism of the effect of different irradiation energy levels on the Hall voltage change will be analyzed next.

**Figure 10:**
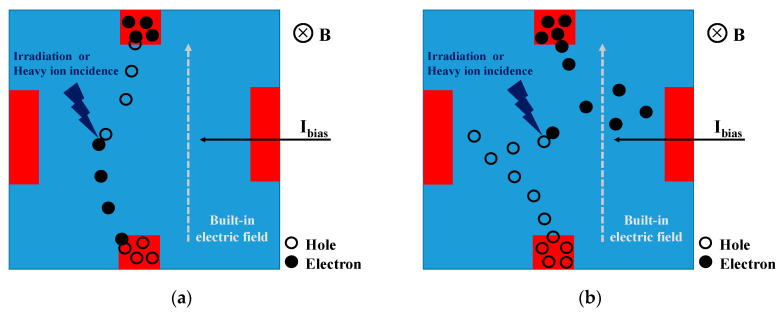
Illustration of the physical mechanism during (**a**) low- and (**b**) high-energy irradiation at the X-Y cross section of the silicon film.

First, under the effect of bias voltage and magnetic induction, a Hall voltage is generated and a built-in electric field is formed in the direction of Hall contacts, as shown in Figure 10a. Secondly, fewer e-h pairs are generated because of the low-energy nuclear explosions or heavy ion incidence. They will move under the action of the built-in electric field, thereby weakening the built-in electric field, which will cause the transient Hall voltage to decrease. Finally, when all the generated e-h pairs are collected, the Hall voltage returns to the initial value.

As for the irradiation in the case of high energy, as shown in Figure 10b, the e-h pairs generated by irradiation still weaken the built-in electric field first, so the transient Hall voltage first decreases. Secondly, because of the high irradiation energy, a mass of e-h pairs are generated, and they mainly move toward the bias contacts. During this process, electrons and holes move toward the Hall contacts under the influence of the Lorentz force. At this time, the built-in electric field will be strengthened, and the transient Hall voltage will increase. Finally, after the irradiation is completed, a new dynamic balance is reached, and the built-in electric field and Hall voltage are slightly larger than the initial value.

Therefore, under different irradiation energies, the two electric fields (the electric field formed by the bias contacts and the built-in electric field formed by the Hall contacts) that dominate the movement of the e-h pairs will determine how the transient Hall voltage changes.

## 4. Conclusions

This work investigated the impacts of the irradiation ionization effects (the TID, TDR, and SET effects) on the performance of the FD-SOI Hall sensors. The sensor FOMs were evaluated in terms of the Hall voltage, sensitivity, and efficiency via the TCAD simulations based on Synopsys Sentaurus^®^. Moreover, the physical mechanism of the effect of irradiation on performance was also analyzed.

For the TID effect, the sensitivity increases, the efficiency decreases, and the offset voltage decreases because of the trapped holes in the oxide layer after irradiation. As for the TDR and SET effects, the transient Hall voltage changes in the off-state are also different due to either nuclear explosion or heavy ion incidence at different energies. This is mainly due to the difference in the moving direction of e-h pairs generated under different irradiation energy under the bias electric field and built-in electric field.

To the authors’ best knowledge, these results comprehensively summarized the response of the FD-SOI Hall sensors in terms of irradiation ionization effect for the first time. These results provide deep insight into the design, processing, and testing of the FD-SOI Hall sensors used in radiation environments.

## Figures and Tables

**Table 1:** The figure-of-merits (FOMs) definitions for Hall sensor ^1^.

FOMs	Relations	References
Hall voltage (*V_H_*)	$V_{H}=G\;\frac{r_{H}}{nqt}I_{bias}B$	[1]
Absolute sensitivity (*S_A_*)	$S_{A\;}=\frac{V_{H}}{B}=G\;\frac{r_{H}}{nqt}I_{bias}$	[1]
Efficiency factor (*η*)	${\;\eta }_{\;}=\frac{S_{A}}{P}=\frac{V_{H}}{V_{bias}{\cdot I}_{bias\;}\cdot B}\;[\frac{V}{W\cdot T}]$	[21]
Offset voltage (*V_offset_*)	N/A	[20]

^1^ where *G* is the geometrical correction factor, *r_H_* is the scattering factor, *I_bias_* is the bias current, *B* is the magnetic field induction, *n* is the carrier density, *q* is the electron charge, *t* is the thickness of the active region, *P* is the dissipated power and *V_bias_* is the bias voltage.

**Table 2:** The FD-SOI Hall sensors design features.

Geometrical Dimensions		Doping Concentrations	
L	15 µm	Silicon film	P-type: N_A_ = 1 × 10^16^ cm^−3^
W	15 µm		
L_a_	1 µm		
W_b_	7.5 µm		
Gate oxide	100 nm	Contacts	N-type: N_D_ = 1 × 10^21^ cm^−3^
Silicon film	50 nm		
Buried oxide	400 nm		
Substrate	5 µm		

**Table 3:** Radiation effects on performance of FD-SOI Hall sensor ^1^.

Effects	Time	Changed Values	Effect on Performance					
			Change	*I_bias_*	*V_H_*	*S_A_*	*η*	*V_offset_*
total ionizing dose (TID)	After irradiation	Total dose	↑	↑	↑	↑	↓	↓
					**Transient *V_H_***			
transient dose rate (TDR)	During irradiation	Dose rate	↑	↑	↘↗			
single event transient (SET)		LET						

^1^ ↑ and ↓ indicate an increase or decrease in value while ↘↗ indicates that the value decreases first and then increases.

## Appendix A

This appendix briefly summarizes the previous work of [8] and focuses on the selection of the optimal sensor structure. Compared with the bulk structure, the characteristics of the FD-SOI Hall sensors (such as sensitivity and efficiency) are improved because of its thin thickness and low doping concentration.

If the FD-SOI structure is to be obtained, the thickness of the silicon film needs to be less than twice the thickness of the maximum depletion layer (*X_dmax_*) under the gate oxide. The *X_dmax_* is determined as follows:

(A1) $$X_{dmax}=\sqrt{\frac{{\;4\varepsilon }_{si}\varphi_{F}}{{qN}_{A}}}$$

where *N_A_* is the doping concentration of the silicon film, *ε_si_* is the dielectric constant of silicon, and *φ_F_* is the Fermi potential. Therefore, the silicon film thickness of 50 nm matches the doping concentration of 10^16^ cm^−3^, which can make the sensor reach FD-state.

In addition to FD-state, other structural features also play an important role in improving the characteristics of the sensor. High-symmetry square sensors demonstrate the best efficiency, which is the best trade-off between sensitivity and power consumption. The shorter length (1 μm) of the Hall contacts and the width of the 0.5× W bias contacts can improve the sensitivity of the sensor.
